# Conjunctival Metastasis as an Initial Sign of Small Cell Lung Cancer

**DOI:** 10.1155/2014/614353

**Published:** 2014-10-23

**Authors:** Afsun Sahin, Nilgun Yildirim, Deniz Goren Sahin, Hikmet Basmak, Mustafa Acikalin

**Affiliations:** ^1^Department of Ophthalmology, Eskisehir Osmangazi University Medical School, 26480 Eskisehir, Turkey; ^2^Department of Ophthalmology, Eskisehir Osmangazi University Hospitals, Meselik, 26480 Eskisehir, Turkey; ^3^Department of Internal Medicine, Eskisehir Osmangazi University Medical School, 26480 Eskisehir, Turkey; ^4^Department of Pathology, Eskisehir Osmangazi University Medical School, 26480 Eskisehir, Turkey

## Abstract

*Introduction*. To report a case of a conjunctival metastasis as the initial manifestation of small cell lung cancer. *Methods*. Observational case report. *Results*. A 50-year-old man without known systemic disease developed a conjunctival mass in his right eye. He underwent incisional biopsy of the tumor and systemic evaluation. Histopathologically, the conjunctival mass was a metastatic small cell carcinoma. Further evaluation revealed a primary small cell lung carcinoma with metastasis to liver and bones. The primary and metastatic tumors were treated with chemotherapy. *Conclusion*. Conjunctival metastasis may be the initial manifestation of lung cancer. It should be considered in the differential diagnosis of a deep conjunctival mass.

## 1. Introduction

Metastatic neoplasms to the conjunctiva are extremely rare. However, they have arisen from cancer of breast, lung, and elsewhere, including cutaneous melanoma [1–4]. Conjunctival metastasis usually occurs as a part of widespread metastatic disease [1, 2]. Rarely metastasis can be the presenting feature with no evidence of any systemic malignancy [3–5].

Ocular metastasis from lung carcinoma is a well-recognized phenomenon. Small cell lung carcinoma constitutes about 15–23% of the all lung neoplasias. It may present as metastases without any visible primary tumor and is clinically more aggressive [3, 5]. Herein, we present a case of conjunctival mass in a patient who was subsequently diagnosed as having small cell carcinoma of the lung.

## 2. Case Report

**Figure 1:**
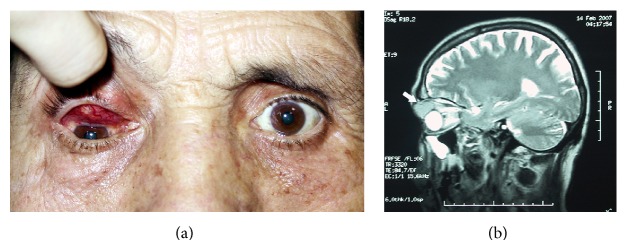
(a) Clinical appearance of the conjunctival mass lesion. Note that the globe is significantly displaced inferiorly. (b) Magnetic resonance imaging of the conjunctival mass lesion (arrow).

A 50-year-old man was referred with a 3-week history of an enlarging mass lesion on his right eye. His medical history was significant only for intense cough. His vision was 20/20 in each eye, and a complete ocular examination revealed normal findings, except for a mass lesion involving the superior bulbar conjunctiva of the right eye and significantly displacing the globe inferiorly (Figure 1(a)). The mass was hard, immobile, and significantly vascularized. There were engorged vessels covering the mass and the color was reddish. The diagnostic impression on clinical and radiological findings was of a probable metastatic neoplasm (Figure 1(b)). He underwent incisional biopsy of the tumor and systemic evaluation. Systemic evaluation revealed previously unsuspected right hilar mass, liver metastasis, and bronchoscopy revealed widespread cancerous invasion of the carina. An incisional biopsy of the carina was also performed.

**Figure 2:**
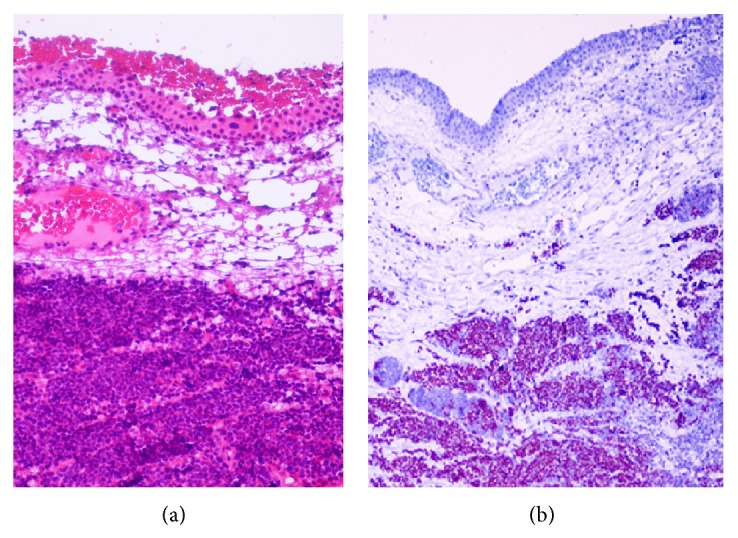
(a) Photomicrograph showing cellular neoplasm with darkly staining nuclei and scanty cytoplasm in the subepithelial stroma of the conjunctiva (stain, hematoxylin-eosin; original magnification, ×200). (b) Photomicrograph showing intense immunoreactivity to thyroid transcription factor- (TTF-) 1, consistent with small cell lung cancer (stain, TTF-1; original magnification, ×100).

Histopathologic evaluation of the conjunctival tumor disclosed a neoplasm that was located in the conjunctival stroma. The highly cellular tumor was composed of small round to oval shaped cells with scant cytoplasm, finely granular chromatin, and absent or inconspicuous nucleoli, compatible with metastatic small cell lung cancer (Figure 2(a)). The mitotic count was high. The tumor cells showed intense immunoreactivity to thyroid transcription factor- (TTF-) 1 and keratin, consistent with small cell lung cancer (Figure 2(b)). The small round to oval shaped cells that comprised the tumor and its location beneath the conjunctival epithelium, without an epithelial or junctional component, supported the diagnosis of the metastatic small cell lung cancer. Histopathologic evaluation of the specimen from the carina further revealed small cell lung carcinoma.

## 3. Discussion

The conjunctiva is rarely the site of metastatic neoplasias [1]. Metastasis to the conjunctiva from primary lung cancer is uncommon and patients such as this are extremely rare [2–5]. Kiratli et al. [2] reported 10 patients with conjunctival metastasis. Of the 10 cases, the primary neoplasm was breast cancer in 4, lung cancer in 2, cutaneous melanoma in 2, laryngeal carcinoma in 1, and unknown in 1.

The patient reported here developed conjunctival metastasis as the initial sign of the primary lung cancer. Shields et al. [3] reported a similar patient to ours in whom conjunctival metastasis was the first presentation of the lung cancer. However, the histological type of the tumor was a squamous cell carcinoma.

Our case demonstrates that conjunctival metastasis from primary small cell lung cancer can be the first sign of disseminated disease. Even though the patient reported here was subsequently found to have liver and bone metastases, the conjunctival metastasis was clearly the initial sign of the metastatic disease. Conjunctival metastasis of small cell lung cancer is rare, and it is clinically difficult to differentiate eyelid tumors as benign or concerning by examination alone. This case highlights the importance of a thorough history and biopsy to diagnose a metastatic neoplasm in a patient at high risk for cancer. The clinicians should be aware of metastatic lung cancer in the presence of a conjunctival mass lesion.
